# Effect of Ephedrae Herba methanol extract on high-fat diet-induced hyperlipidaemic mice

**DOI:** 10.1080/13880209.2019.1666883

**Published:** 2019-09-23

**Authors:** Se-Eun Lee, Chiyeon Lim, Sehyun Lim, Byoungho Lee, Suin Cho

**Affiliations:** a Department of Korean Medicine, School of Korean Medicine, Pusan National University, Yangsan, Gyeongnam, Republic of Korea;; b Department of Medicine, College of Medicine, Dongguk University, Republic of Korea;; c Department of Nursing Science, School of Public Health, Far East University, Chungbuk, Republic of Korea

**Keywords:** Obesity, cardiovascular diseases, weight reduction, herbal medicine

## Abstract

**Context:** Ephedrae Herba (EH), the dried stems and leaves of *Ephedra sinica* Stapf., *E. intermedia* Schrenk et C. A. Mey., or *E. equisetina* Bge. (Ephedraceae [Ephedra]) is used to treat respiratory diseases. Recently, especially in the Republic of Korea, EH has also been used for weight reduction.

**Objective:** We evaluated the effects and molecular targets of methanol EH extract (EHM) on high-fat diet (HFD)-induced hyperlipidemic ICR mice.

**Materials and methods:** EHM was orally administered (100 mg/kg body weight/day) for 3 weeks. We observed changes in body weight (BW), total cholesterol (TC), high-density lipoprotein–cholesterol, and triglycerides to evaluate the physiological changes induced by HFD or EHM administration. To evaluate lipid peroxidation and liver toxicity, malondialdehyde and blood alanine aminotransferase levels were measured. In addition to analyzing liver gene expression profiles, EHM target proteins were identified using a protein interaction database.

**Results:** EHM administration for 3 weeks significantly (*p* < 0.05) decreased TC and triglyceride levels without altering BW in mice, and gene expression levels in the livers of EHM-treated mice were restored at 34.0% and 48.4% of those up- or down-regulated by hyperlipidaemia, respectively. Proteins related to DNA repair and energy metabolism were identified via protein interaction network analysis as molecular targets of EHM that play key roles in ameliorating hyperlipidaemia.

**Discussion and conclusions:** EHM regulated hyperlipidaemia by decreasing total blood lipid and triglyceride levels in hyperlipidaemic mice. EHM showed preventive effects against hyperlipidaemia in mice, possibly via the regulation of DNA repair and the expression of energy metabolism-related genes and proteins.

## Introduction

In modern society, the prevalence of various metabolic disorders such as diabetes, hypertension, hyperlipidaemia, and cardiovascular diseases is increasing as a result of drastic changes in the living environment and excessive nutritional intake (Bao et al. 2017; Danese et al. 2017). Among the main causes of death in Korea, cerebrovascular and cardiac diseases are listed as second and third, respectively, after cancer (Lee et al. 2016; Yun and Son 2016; Kim et al. 2017).

Hyperlipidaemia is closely related to the intake of food rich in cholesterol and triglycerides. In particular, excessive intake of animal-derived saturated fat is reported to increase total cholesterol (TC) concentrations in the serum and can cause various diseases, including cardiovascular diseases (Kopelman 2000; Jelcic and Korsic 2009; Efremov et al. 2013; Mattar et al. 2017). Thus, hyperlipidaemia is both a threat to human health and a social concern (Jelcic and Korsic 2009; Efremov et al. 2013).

Hyperlipidaemia is recognized as a direct cause of cerebrovascular and heart diseases; therefore, therapeutic methods for controlling hyperlipidaemia have been widely studied (Sanders et al. 1997). However, because of the risk of adverse effects associated with long-term use of medication, there is an increasing tendency to prefer diets that include natural products with high efficacy against hyperlipidaemia with few side effects (Yao and MacKenzie 2010).

The traditional medicine industry has gained interest from the pharmaceutical industry through the development of new drugs. Therefore, in this study, we assessed the activity of an herbal medicine extract and investigated new molecular targets of Ephedrae Herba (EH) in hyperlipidaemic mice. Recently, several studies have reported that medicines containing EH were effective in weight reduction (Shin and Yoon 2012; Fan et al. 2015; Roh et al. 2017; Lim et al. 2018), but there are few studies regarding the effects of EH alone, and the associated underlying mechanisms.

Traditionally, EH has mainly been used to treat symptoms of respiratory diseases such as asthma, although recently, it has increasingly been used for weight reduction purposes (Shin and Yoon 2012; Fan et al. 2015). In the Republic of Korea, EH is derived from the dried stems and leaves of *Ephedra sinica* Stapf., *E. intermedia* Schrenk et C. A. Mey., or *E. equisetina* Bge. (Ephedraceae [Ephedra]), and has been widely used for the treatment of asthma and coughs, and as a diaphoretic (Herbology Editorial Committee of Korean Medical Schools 2012).

The reported effects of EH are highly diverse and include antioxidant (Okawa et al. 2001), anti-inflammatory (Aoki et al. 2005), antimicrobial (Zang et al. 2013), antidiabetic (Xiu et al. 2001), antiasthmatic (Chu et al. 2006; Liu and Luo 2007), antimelanogenic (Kim et al. 2006), and antiobesity (Shin and Yoon 2012; Fan et al. 2015; Roh et al. 2017; Lim et al. 2018) activities. A recent report related to the present study investigated the effect of *E. sinica* extracts on hyperlipidaemia in mice (Fan et al. 2015). The authors orally administered ephedra alkaloids, ephedra polysaccharides, and ephedra non-alkaloids separately to mice and concluded that ephedra non-alkaloids showed therapeutic potential for the treatment of hyperlipidaemia in mice (Fan et al. 2015).

The best known major bioactive constituents of EH are ephedrine-type alkaloids (ephedrines), which act as sympathomimetics by stimulating the heart rate and promoting broncho-dilatation (Chang et al. 2018; Han et al. 2018). However, food supplements containing ephedrines also represent substantial health risks, and consequently, many countries have instituted bans of all over-the-counter drugs containing ephedrine (Han et al. 2018; Sellami et al. 2018).

The side effects of EH are well-known and have been described in many studies; however, in Korea, because of its weight loss effects, many traditional Korean medicine practitioners continue to prescribe EH for patients with hyperlipidaemia and obesity (Shin and Yoon 2012; Lim et al. 2018).

Through our preliminary study, we confirmed that the methanol extract of EH (EHM) was effective in reducing blood cholesterol without any adverse effects. In the present study, the effects and molecular targets of EHM in high-fat diet (HFD)-induced hyperlipidaemic mice were investigated. Changes in body weight (BW), total cholesterol (TC) content in the serum, high-density lipoprotein-cholesterol (HDL-C), and triglycerides were measured to evaluate the antihyperlipidaemic effects of EHM. Lipid peroxide accumulation due to lipid metabolism disorders was also evaluated by measuring malondialdehyde (MDA) levels. In addition, using a protein interaction database, the target proteins of EHM were identified via gene expression analysis in hepatic tissues.

## Materials and methods

### Animals

To induce hyperlipidaemia, 6-week-old male ICR mice (Samtako, Korea) weighing 20 to 25 g were used. All the mice used in this experiment were obtained from a specific pathogen-free (SPF) barrier facility, and were adapted to laboratory environment (room temperature of 24 ± 2 °C, humidity 55 ± 5%, 12 h dark/light cycles) for 1 week or more while supplying sufficient solid feed and water. The animal experiment protocol was approved by the ethics committee of Pusan National University (approval number PNU-2013-0311).

### EHM preparation

**Figure 1.**
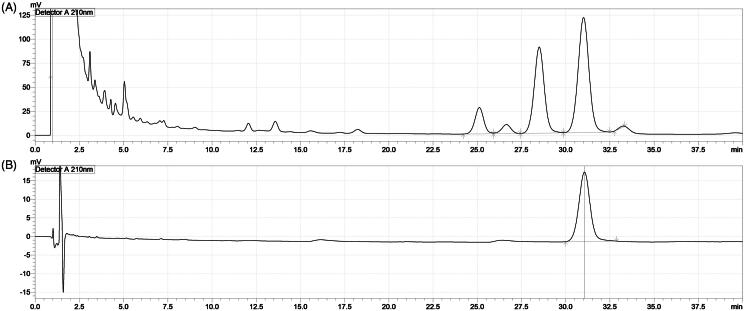
High-performance liquid chromatography (HPLC) chromatograms of Ephedrae Herba methanol extract (EHM) and its active compound, ephedrine. A, the chromatogram of EHM; B, chromatogram of ephedrine. HPLC, Shimadzu; pump, LC-20AD; auto-sampler, SIL-20A; detector, SPD-M20A; column oven, CTO-20A. Mobile phase, mixture of sodium lauryl sulphate, acetonitrile, and phosphoric acid (640:360:1). Column, YMC-Triart C18, 250 × 4.6 mm, 5 μm; column temperature, 45 °C; flow rate, 1.5 mL/min; injection volume, 10 μL.

EH was purchased from an authorized pharmaceutical company (Naemomedah Co., Korea) and authenticated as originating from *E. sinica.* A voucher specimen (no. EH14-0217) was deposited in the low-temperature room (4 °C) of the laboratory. Dried EH (500 g) was immersed in methanol at room temperature for 5 days, and the process used to obtain the filtrate was repeated twice. The resulting lyophilized EHM extract weighed 41.4 g, and the yield was 8.3%. Many of the effects of EH are considered to be associated with ephedrine (Chang et al. 2018); therefore, we detected the presence of ephedrine to obtain a fingerprint of the EHM used in this experiment (Figure 1).

### Induction of hyperlipidaemia and classification of experimental groups

**Figure 2.**
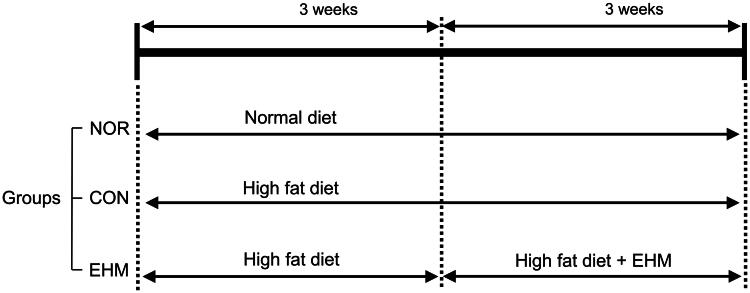
Hyperlipidaemia induction in mice and EHM administration. The mice were fed a normal or high-fat diet (HFD) for 6 weeks before group assignment. EHM was orally administered to the EHM group for the last 3 weeks. NOR: normal diet-fed control mice (*n* = 8), CON: HFD-fed hyperlipidaemic mice (*n* = 8), EHM: HFD-fed and EHM-administered mice (*n* = 8).

To induce hyperlipidaemia in mice, mice in the control group (CON) and EHM-treated group (EHM) were fed a HFD for 3 weeks, and mice in the normal group (NOR) were supplied general feed. In week 4 of the experiment, HFD-fed mice were randomly assigned to the CON and EHM groups based on their BWs. Therefore, from week 4, EHM group mice were fed a HFD with orally administered EHM (100 mg/kg BW/day), while the CON group mice were fed the same diet and received the same volume of vehicle administered orally for a further 3 weeks. The dose was optimized through our preliminary study in which EHM showed no observed adverse effect level (NOAEL). Thus, no LD50 was applied to this study. The rodent chow used was manufactured by Daol Biotech (Daejeon, Korea), and its composition is shown in Table S1. A schematic representation of the study design is shown in Figure 2. The BW of the mice was measured every two weeks during the experiment.

### Blood collection and harvesting liver tissues

After euthanizing the mice, blood was collected via the abdominal vein and the liver tissue was excised after trans-cardiac perfusion with ice-cold (4 °C) perfusion solution containing 130 mM NaCl, 5 mM KCl, and 10 mM Tris-HCl (pH 7.4). Whole blood was centrifuged at 5000 *×* *g* for 20 min to obtain the serum, and the supernatant was collected for measurement of blood cholesterol and triglyceride content.

### Liver tissue preparation for lipid peroxidation evaluation and gene expression analysis

Lipid peroxidation, which refers to the oxidative degeneration of lipids, was evaluated by measuring MDA, one of the oxidative end-products (Gutteridge 1995). A Stadie-Riggs microtome (Thomas Scientific, Swedesboro, NJ, USA) was used to prepare tissue slices approximately 1 mm wide and 0.4–0.5 mm thick with horizontal and vertical lengths of 1 cm each to measure MDA content. Phosphoric acid (3 mL) and 0.6% thiobarbituric acid solution were added, and the solution was boiled for 60 min, before adding 4 mL 1-butanol, mixing, and centrifuging at 800 *×* *g* for 25 min. Finally, the absorbance of the supernatant was measured at 534 and 510 nm.

To evaluate gene expression, total RNA was isolated using a Qiagen RNeasy Kit (Qiagen, Hilden, Germany) according to the manufacturer’s instructions, and an Agilent microarray containing approximately 45,000 oligo-spots (Agilent Technologies, Santa Clara, CA, USA) was used for hybridization. RNA from NOR group mice was used as a reference, and a change in expression from the baseline ≥2-fold was considered as up- or down-regulation. Hierarchical clustering of genes was performed using a multiple experiment viewer (MeV ver. 4.9, mev.tm4.org), and a functional protein association networks database (STRING Consortium 2003) was used for interaction network analysis.

### Measurement of blood cholesterol, HDL-C, triglycerides, and alanine aminotransferase (ALT)

Blood TC, HDL-C, and triglyceride content were measured using a measurement kit (Fujifilm, Tokyo, Japan), and ALT was determined spectrophotometrically using a direct reading assay kit (Asan Pharmaceutical Co., Seoul, Korea).

### Statistical analysis

The statistical package SigmaPlot ver. 12 (Systat Software, San Jose, CA, USA) was used for analysis. Experimental results are expressed as means ± standard deviation (SD), and statistical significance among the groups was determined via one-way analysis of variance (ANOVA), followed by Tukey’s *post hoc* analysis, using SigmaPlot. Statistical significance was ascribed when *p* < 0.05.

## Results

### Effects of EHM on body weight and blood lipid content of mice

**Figure 3.**
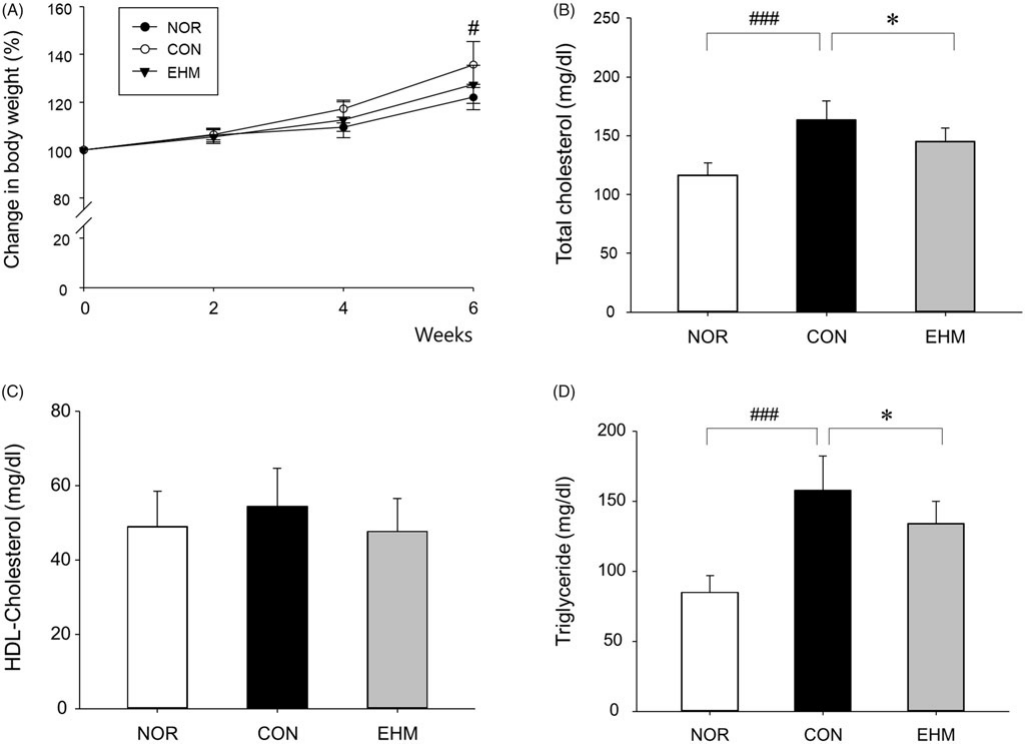
Effects of EHM on body weights, blood levels of total cholesterol, high-density lipoprotein (HDL)-cholesterol, and triglycerides in hyperlipidaemic mice. Body weights (A) were measured every 2 weeks. The levels of total cholesterol (B), HDL-cholesterol (C), and triglycerides (D) in blood serum were determined via spectrophotometry. NOR: normal diet-fed control mice (*n* = 8), CON: HFD-fed hyperlipidaemic mice (*n* = 8), EHM: HFD-fed and EHM-administered mice (*n* = 8). Values are presented as the means ± standard deviation (SD). ^###^*p* < 0.001 compared to the NOR group; *p < 0.05 compared to the CON group.

Compared with the normal diet-fed NOR group, 6-week HFD-fed mice showed significant (*p* < 0.05) BW gain, although no significant difference was observed when comparing mice from the EHM and CON groups (Figure 3(A)). Furthermore, no differences in food intake were observed among the different groups during the experimental period (data not shown).

TC content in mouse blood was significantly higher (*p* < 0.001) in the CON group than that in the NOR group (163.6 ± 16.1 mg/dL vs 116.4 ± 10.5 mg/dL, respectively). Furthermore, TC was significantly lower (*p* < 0.05) in the EHM group (145.3 ± 11.5 mg/dL) than in the CON group (Figure 3(B)). No significant differences were observed regarding the HDL-C content across the different groups (Figure 3(C)). A statistically significant increase in blood triglyceride levels was observed in the CON group (85.1 ± 11.7 mg/dL) compared with that in the NOR group (158.0 ± 24.2 mg/dL, *p* < 0.001) and in the EHM group (134.3 ± 15.8 mg/dL, *p* < 0.05; Figure 3(D)).

### Effects of EHM on liver MDA content and blood ALT content

**Figure 4.**
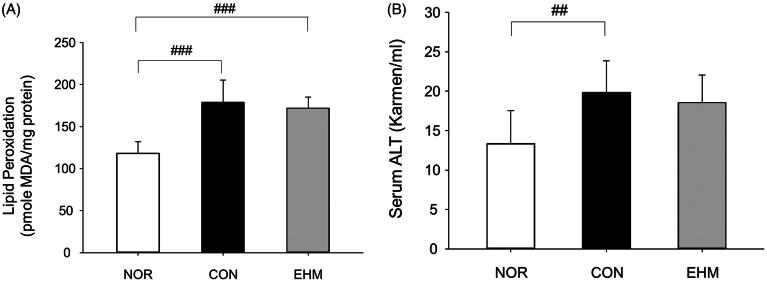
Effects of EHM on lipid peroxidation and blood alanine aminotransferase (ALT) levels in hyperlipidemic mice. Lipid peroxidation in liver tissues and blood ALT levels were determined via spectrophotometry. NOR: normal diet-fed control mice (*n* = 8), CON: HFD-fed hyperlipidaemic mice (*n* = 8), EHM: HFD-fed and EHM-administered mice (*n* = 8). Values are presented as means ± SD. ^##^*p* < 0.01 and ^###^*p* < 0.001 compared to the NOR group.

MDA content was significantly higher in the CON group (178.8 ± 26.9 pmol MDA/mg protein) than that in the NOR group (118.1 ± 14.0 pmol MDA/mg protein, *p* < 0.001). Interestingly, the EHM group also showed a significant increase (*p* < 0.001) in liver MDA content (172.1 ± 13.2 pmol MDA/mg protein) compared with that in the NOR group (Figure 4(A)), although EH was previously reported to have antioxidant effects (Okawa et al. 2001). Blood ALT levels in the CON group were significantly higher (*p* < 0.01) than those in the NOR group, but no significant difference was observed when comparing levels in the EHM group to those of other groups (Figure 4(B)).

### Effects of EHM on liver gene expression

**Figure 5.**
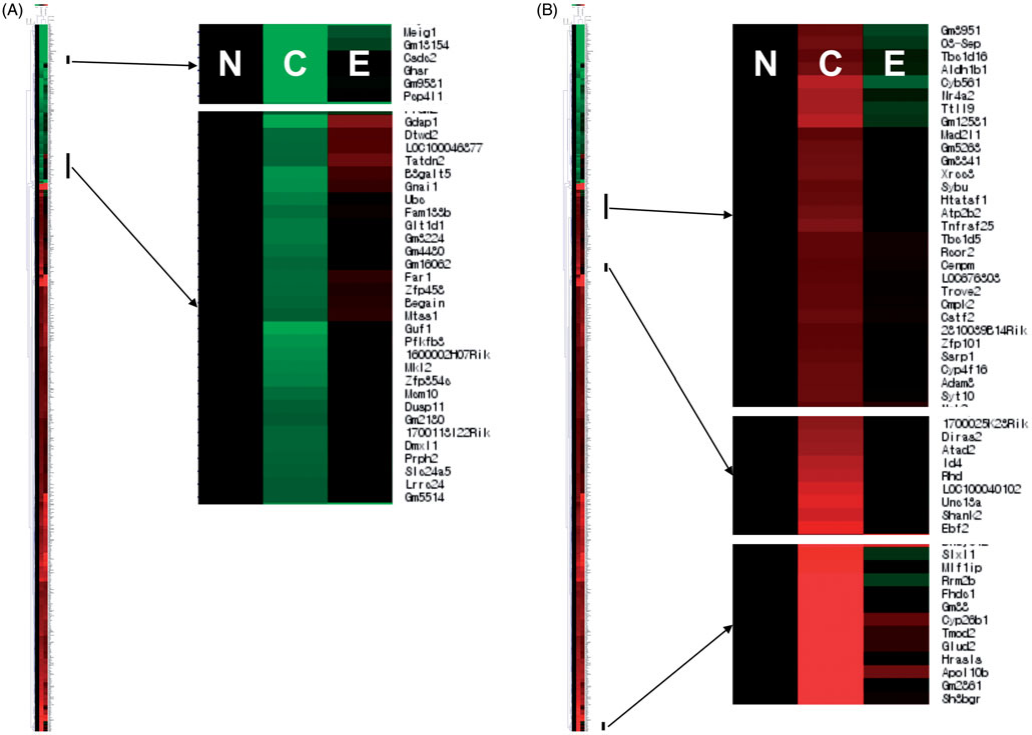
Effects of EHM on the expression of genes in the livers of hyperlipidaemic mice. To identify genes via quantitative analysis and hierarchical clustering, MeV software ver. 4.0 was used. Red indicates genes showing ≥2-fold up-regulated expression compared to that in the NOR group; green indicates genes showing ≥2-fold down-regulated expression compared with that in the NOR group. N: normal diet-fed control mice (NOR group), C: HFD-fed hyperlipidaemic mice (CON group), E: HFD-fed and EHM-administered mice (EHM group).

**Figure 6.**
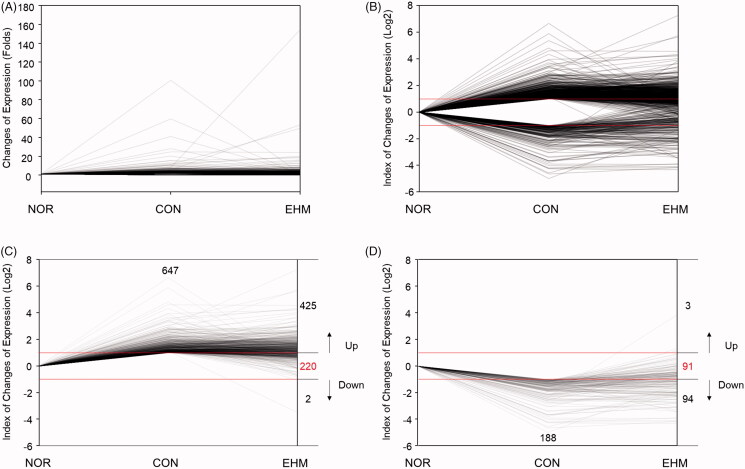
Line plot of alterations in liver gene expression in hyperlipidemic mice. Raw fold values (A) for gene expression compared with that in the NOR group were converted to log values (B) for each differentially expressed gene, and the numbers of up-regulated (C), down-regulated (D), and restored (C, D) genes were reported. NOR: normal diet-fed control mice, CON: HFD-fed hyperlipidemic mice, EHM: HFD-fed and EHM-administered mice.

Gene expression patterns in the livers of mice were observed, and a total of 835 genes showing ≥2-fold variations in expression in the CON group compared with that in the NOR group were hierarchically clustered (Figure 5). The expression of these genes was significantly changed in the livers of mice in the CON group compared with those of the NOR group. Among the altered genes, we selected 220 down-regulated and 91 up-regulated genes whose expression was restored by EHM administration based on hierarchical clustering using MeV software (Figure 5). Detailed alteration and restoration of gene expression are shown in Figure 6.

**Figure 7.**
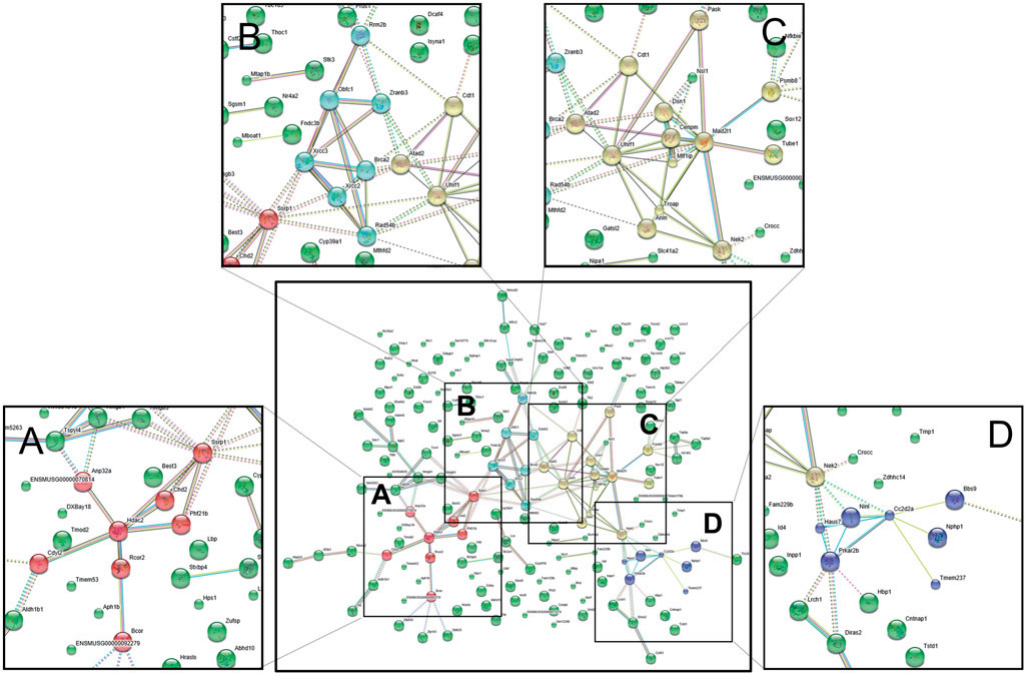
Protein network analysis using STRING data resources. Information regarding the restoration of gene expression after EHM administration in hyperlipidemic mice was uploaded to the STRING database (version 11.0) to investigate interactions between related proteins. Network nodes represent proteins, edges represent protein–protein interactions, and the line thickness indicates the strength of data support. Four main protein–protein interactions were observed, and Hdac2 (A), Xrcc2-Xrcc3 (B), Uhrf1 (C), and Prkar2b (D) were identified as key proteins in these interactions.

We identified the main target proteins predicted to play key roles (Figure 7) using functional protein association network databases such as the STRING database (STRING Consortium 2003) to explore predicted protein interaction networks and suggest new directions for future experimental research by assessing 311 genes whose expression was restored by EHM administration.

## Discussion

Recently, the incidence of obesity has increased at an alarming rate, thus causing many public health concerns. The westernization of dietary habits has caused a steady increase in the occurrence of coronary artery disease, which is a cause of hyperlipidemia, and total blood lipid content elevation related to abnormal lipid metabolism in the body (Bao et al. 2017; Danese et al. 2017). In Western medicine, 3-hydroxy-3-methylglutaryl coenzyme A reductase inhibitors (statins) have been used effectively in patients with primary or secondary ischaemic heart disease, hypercholesterolaemia, and cardiovascular disease, although various side effects have been reported (Anyanwagu et al. 2017). The most common adverse reaction is myalgia, which affects approximately 10% of patients with myasthenia. Digestive disorders, heartburn, and abdominal pain occur in approximately 4% of the cases, and elevated liver enzyme levels have also been reported (Kashani et al. 2008). Considering the side effects of statins, many studies have been conducted to develop drugs for the treatment of hyperlipidaemia using alternative medicine, complementary medicine, and natural products. Recently, various natural bioactive compounds considered safe and effective, such as curcumin, propyl gallate, and resveratrol, have been explored for the treatment of obesity (WHO 2018; Feng et al. 2019).

EH, the dried aerial parts of *E. sinica*, has been widely used in Asian traditional medicine to treat bronchial asthma, cold, fever, headache, and cough (Han et al. 2018). Recently, and especially in the Republic of Korea, EH has been used in antiobesity prescriptions in traditional medicine clinics (Shin and Yoon 2012; Roh et al. 2017; Lim et al. 2018), although its toxicity and mechanism of action remain unclear.

Ephedrine is an alkaloid with ergogenic properties that can be found in EH, and many studies have shown the potential effects of ephedrine on the promotion of increased physical performance and weight loss (Chang et al. 2018; Sellami et al. 2018). Han et al. reported that the NOAEL of an EH water extract was 125 mg/BW/day for rats under experimental conditions (Han et al. 2018), suggesting that EH shows relatively high toxicity compared with other herbal medicines.

In Korean traditional medicine, approximately 10 g of EH is administered to humans daily (Herbology Editorial Committee of Korean Medical Schools 2012); in this experiment, 7.2 times this dose of EH was administered to mice compared to humans. However, considering the metabolic rates of humans and mice, this dose is not considered too high (Han et al. 2018). In a preliminary study, we tested the aqueous and methanol extracts of EH in hyperlipidaemic mice at doses ranging from 10–300 mg/kg BW/day. A dose of 100 mg/kg BW/day (methanol extract) was shown to be effective and safe (below the NOAEL of EHM), and this is the concentration we used here.

In this study, EHM administration significantly decreased (*p* < 0.05) TC and triglyceride levels without altering BW in mice (Figure 3(B,D)). Fan et al. (2015) previously reported lower levels of blood TC and triglycerides after administration of ephedrine or non-alkaloids purified from EH to mice. The authors also reported lower blood ALT levels and liver MDA content after administration of non-alkaloids purified from EH to mice, although in the present study, no changes were observed in ALT levels and MDA contents in the EHM group compared to those in the CON group (Figure 4). ALT is found primarily in the liver and is considered a sensitive indicator of liver damage, and MDA content in liver tissue homogenate was measured to evaluate the antioxidant capacity of EHM (Dawn-Linsley et al. 2005; Ruan et al. 2013). When EHM was administered to mice at a dose of 300 mg/kg, blood ALT levels increased significantly compared to those in the CON group, suggesting that high doses of EHM are likely to cause liver damage (data not shown).

Although underlying antihyperlipidaemic mechanism is not clear, our preliminary study showed a slight absence of hepatic sinusoids following administration of a high-fat diet, and recovery was observed following administration of EHM (Figure S1). There is also a possibility that the occurrence and recovery of these structures may have affected lipid metabolism, resulting in changes in blood lipids.

Liver gene expression profiles of the CON and EHM groups are shown in Figure 5. The threshold for up- and down-regulation was ≥2-fold as determined via microarray analysis, and representative genes whose expression was altered by EHM administration were classified via hierarchical heat map analysis (Figure 5). Although the expression of many genes in the CON group mice was up- (647 genes) or down-regulated (188 genes) compared with that in the NOR group mice, the expression of certain genes was restored in the EHM group (Figure 6). The expression of 34.0% (220/647) and 48.4% (91/188) of up- and down-regulated genes, respectively, was recovered by treatment with EHM (Figure 6).

Using the protein network database STRING, we also identified important target proteins regulated by EHM, and the main protein interactions are presented in Figure 7. Histone deacetylase (Hdac) is an enzyme that is encoded by the Hdac gene in humans, and this gene is reportedly inhibited by statins (Lin et al. 2008). In the present study, Hdac2 was regulated by EHM administration in hyperlipidemic mice (Figure 7(A)), suggesting that EHM will have effects similar to those of statins. In humans, X-ray repair cross-complementing (Xrcc) and ubiquitin-like with PHD and RING finger domains 1 (Uhrf1) proteins play important roles in DNA repair processes, and especially in double-strand break repair (Thacker and Zdzienicka 2003; Hahm et al. 2019), and proteins Xrcc2, Xrcc3, and Uhrf1 were regulated by EHM administration in hyperlipidaemic mice (Figure 7(B,C), respectively). The protein kinase cAMP-dependent regulatory type-II beta (Prkar2b) gene is reportedly involved in energy metabolism and weight gain (Gagliano et al. 2014), and the results of the present study suggest the potential regulatory activity of EHM in hyperlipidemia (Figure 7(D)). Interestingly, metabolic pathway-related gene ontology (GO) terms were also identified by GO enrichment analysis (Table S2).

Considering the above results, EHM, the methanol extract of the dried aerial parts of *E. sinica*, suppressed hyperlipidaemia by regulating serum levels of total cholesterol and triglycerides in HFD-induced hyperlipidaemic mice. In addition, EHM inhibited hyperlipidaemia by restoring the expression of genes and proteins related to DNA repair and energy metabolism, and proteins such as Hdac2, Xrcc2, Xrcc3, Uhrf1, and Prkar2b were identified as molecular targets playing key roles in ameliorating hyperlipidaemia.

## Conclusions

Oral administration of EHM for 3 weeks significantly reduced blood TC and triglyceride levels, and the up- and down-regulated expression of 34.0 and 48.4% of genes by hyperlipidaemia, respectively, was restored by EHM administration. EHM may exert a potential preventive effect against HFD-induced hyperlipidaemia in mice, possibly via the regulation of DNA repair and the expression of energy metabolism-related genes and proteins.

## Supplementary Material

Supple_Figure_S1.docx

Supple_Tables.docx
