# Supplementary material for: Effect of Ephedrae Herba methanol extract on high-fat diet-induced hyperlipidaemic mice
Source: Pharm Biol. 2019 Sep 23;57(1):676–83. doi: 10.1080/13880209.2019.1666883 (PMC6764353; doi:10.1080/13880209.2019.1666883)
Supplement: Supple_Figure_S1.docx [file IPHB_A_1666883_SM2949.docx]

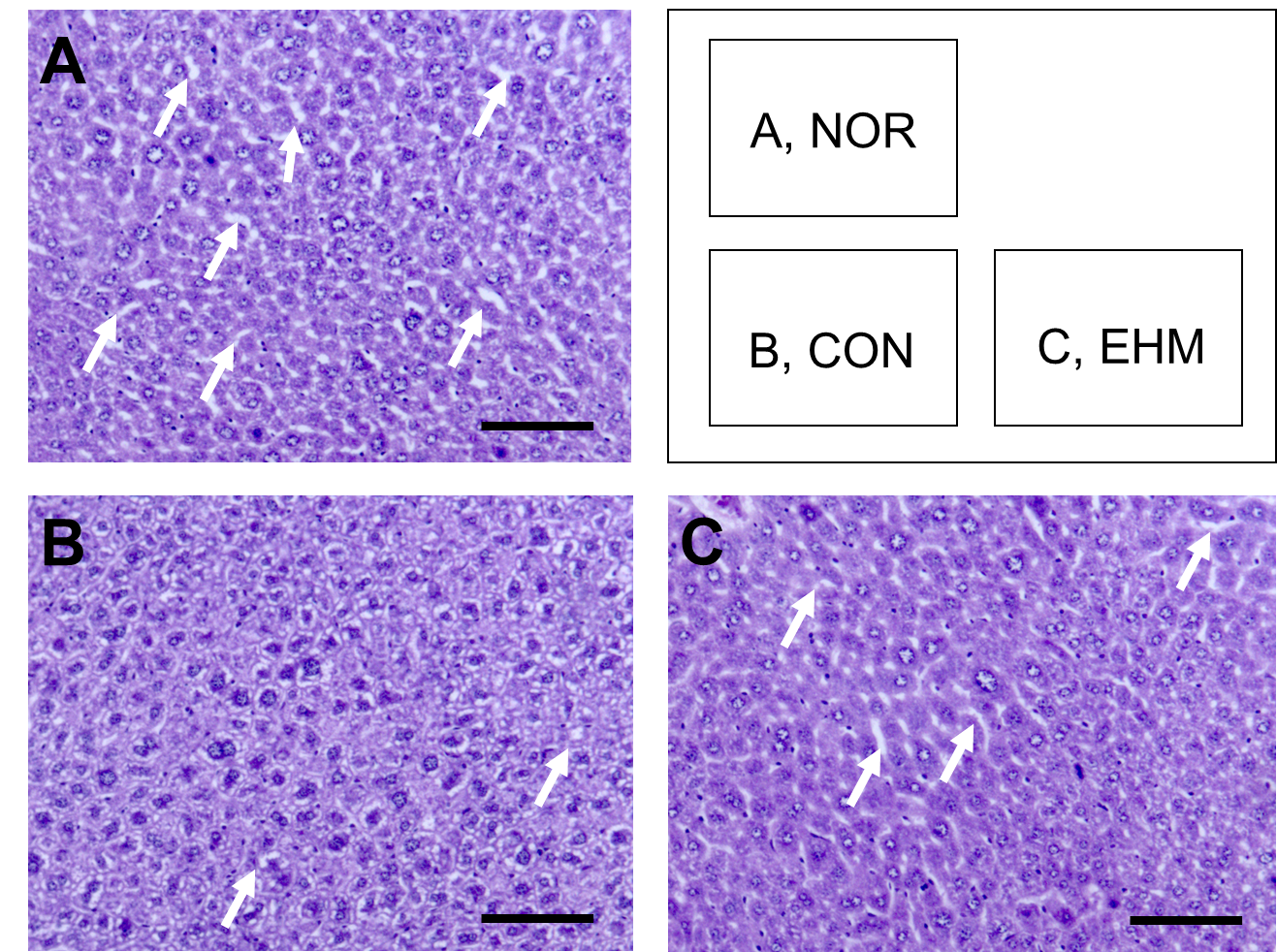


**Figure S1. Pathological section from mice liver of high-fat diet-induced hyperlipidemic mice and effect of Ephedra Herba methanol extract.**

Hepatic tissue sections were stained with hematoxylin and eosin. A, NOR group; B, CON group; C, EHM group. CON group showed a slight absence of sinusoids (white arrows) following administration of a high-fat diet, and recovery was observed following administration of EHM. Scale bar, 100 µm.
