# Supplementary material for: Effect of Ephedrae Herba methanol extract on high-fat diet-induced hyperlipidaemic mice
Source: Pharm Biol. 2019 Sep 23;57(1):676–83. doi: 10.1080/13880209.2019.1666883 (PMC6764353; doi:10.1080/13880209.2019.1666883)
Supplement: Supple_Tables.docx [file IPHB_A_1666883_SM2948.docx]

| Main ingredients | Normal diet | High fat diet |
| --- | --- | --- |
| Casein | 200 | 200 |
| Sucrose | 172.8 | 172.8 |
| Dextrose | 100 | 100 |
| Soybean oil | - | 25 |
| Lard * | - | 177.5 |
| Cholesterol | - | 12.9 |
| Cholic acid | - | 4.3 |

**Table S1. Compositions of the normal and high-fat diets**

*Typical analysis of cholesterol in lard = 0.95 mg/g.

**Table S2. Gene ontology (GO) terms associated with differentially expressed genes in the livers of Ephedra Herba methanolic extract (EHM)-administered hyperlipidemic mice**

| Gene Ontology: Biological process | | | | |
| --- | --- | --- | --- | --- |
| Term ID | Term description | Observed gene count | Background gene count | FDR |
| GO:0009987 | Cellular process | 173 | 12459 | 0.0064 |
| GO:1901360 | Organic cyclic compound metabolic process | 74 | 4057 | 0.0073 |
| GO:0006139 | Nucleobase-containing compound metabolic process | 68 | 3702 | 0.0094 |
| GO:0006725 | Cellular aromatic compound metabolic process | 70 | 3879 | 0.0094 |
| GO:0034641 | Cellular nitrogen compound metabolic process | 75 | 4252 | 0.0094 |
| GO:0046483 | Heterocycle metabolic process | 70 | 3830 | 0.0094 |
| GO:0006974 | Cellular response to DNA damage stimulus | 20 | 619 | 0.0122 |

| Gene Ontology: Molecular function | | | | |
| --- | --- | --- | --- | --- |
| Term ID | Term description | Observed gene count | Background gene count | FDR |
| GO:0000400 | Four-way junction DNA binding | 4 | 18 | 0.0183 |
| GO:0001786 | Phosphatidylserine binding | 5 | 41 | 0.0183 |
| GO:0003676 | Nucleic acid binding | 53 | 2868 | 0.0183 |
| GO:0072341 | Modified amino acid binding | 7 | 80 | 0.0183 |
| GO:0097159 | Organic cyclic compound binding | 81 | 4818 | 0.0183 |
| GO:1901363 | Heterocyclic compound binding | 80 | 4748 | 0.0183 |
| GO:0005488 | Binding | 147 | 10884 | 0.0283 |
| GO:0003677 | DNA binding | 37 | 1897 | 0.0375 |
| GO:0003690 | Double-stranded DNA binding | 21 | 840 | 0.0375 |

| Cellular component | | | | |
| --- | --- | --- | --- | --- |
| Term ID | Term description | Observed gene count | Background gene count | FDR |
| GO:0005622 | Intracellular | 182 | 12462 | 9.26E-07 |
| GO:0044424 | Intracellular part | 180 | 12219 | 9.26E-07 |
| GO:0043226 | Organelle | 165 | 10897 | 9.35E-07 |
| GO:0043229 | Intracellular organelle | 161 | 10645 | 1.87E-06 |
| GO:0044464 | Cell part | 195 | 14017 | 1.87E-06 |
| GO:0005694 | Chromosome | 30 | 929 | 1.68E-05 |
| GO:0043231 | Intracellular membrane-bounded organelle | 138 | 9088 | 5.53E-05 |
| GO:0043227 | Membrane-bounded organelle | 145 | 9775 | 7.56E-05 |
| GO:0005634 | Nucleus | 100 | 6086 | 0.00022 |
| GO:0043232 | Intracellular non-membrane-bounded organelle | 69 | 3809 | 0.00066 |
| GO:0098687 | Chromosomal region | 13 | 285 | 0.0011 |
| GO:0000793 | Condensed chromosome | 11 | 216 | 0.0016 |
| GO:0044427 | Chromosomal part | 23 | 807 | 0.0016 |
| GO:0000779 | Condensed chromosome, centromeric region | 8 | 115 | 0.0021 |
| GO:0005657 | Replication fork | 6 | 61 | 0.0028 |
| GO:0044422 | Organelle part | 113 | 7665 | 0.0034 |
| GO:0000776 | Kinetochore | 8 | 129 | 0.0037 |
| GO:0000777 | Condensed chromosome kinetochore | 7 | 103 | 0.0055 |
| GO:0005737 | Cytoplasm | 137 | 9909 | 0.0062 |
| GO:0000775 | Chromosome, centromeric region | 9 | 186 | 0.0073 |
| GO:0044430 | Cytoskeletal part | 31 | 1460 | 0.0096 |
| GO:0045171 | Intercellular bridge | 5 | 55 | 0.011 |
| GO:0071133 | Alpha9-beta1 integrin-ADAM8 complex | 2 | 2 | 0.0149 |
| GO:0044446 | Intracellular organelle part | 106 | 7416 | 0.0166 |
| GO:0035868 | Alphav-beta3 integrin-HMGB1 complex | 2 | 3 | 0.0222 |
| GO:0044441 | Ciliary part | 13 | 426 | 0.0222 |
| GO:0005874 | Microtubule | 12 | 377 | 0.0225 |
| GO:0042470 | Melanosome | 6 | 103 | 0.0225 |
| GO:0044428 | Nuclear part | 61 | 3798 | 0.0225 |
| GO:0005686 | U2 snRNP | 3 | 18 | 0.0231 |
| GO:0015630 | Microtubule cytoskeleton | 24 | 1106 | 0.0231 |
| GO:0000444 | MIS12/MIND type complex | 2 | 4 | 0.0262 |
| GO:0031981 | Nuclear lumen | 55 | 3386 | 0.0279 |
| GO:0070013 | Intracellular organelle lumen | 61 | 3882 | 0.0319 |
| GO:0033063 | Rad51B-Rad51C-Rad51D-XRCC2 complex | 2 | 5 | 0.032 |
| GO:0099513 | Polymeric cytoskeletal fiber | 15 | 581 | 0.032 |
| GO:0005929 | Cilium | 15 | 587 | 0.0334 |
| GO:0005856 | Cytoskeleton | 35 | 1933 | 0.0338 |

False discovery rate (FDR) corrections were performed using the Benjamini-Hochberg method.
